# Human Infection Caused by *Leptospira fainei*

**DOI:** 10.3201/eid0808.010445

**Published:** 2002-08

**Authors:** Jean-Pierre Arzouni, Philippe Parola, Bernard La Scola, Danièle Postic, Philippe Brouqui, Didier Raoult

**Affiliations:** * Unité des Rickettsies, Université de la Méditerranée, Marseille, France; † Service des Maladies Infectieuses et Tropicales, Marseille, France; ‡ Institut Pasteur, Paris, France

**Keywords:** *Leptospira fainei*, human, Western blot, emerging pathogen, Europe

## Abstract

We report a human case of leptospirosis in which the spirochete was detected by dark-field microscopy examination of cerebrospinal fluid (CSF) and isolated from both CSF and blood. *Leptospira fainei* was identified by sequencing the 16S rDNA gene, which had been amplified by polymerase chain reaction. This case confirms the role of *L. fainei* as a human pathogen and extends its distribution to southern Europe.

 Leptospirosis is a worldwide zoonosis, usually transmitted to humans through contaminated water or direct exposure to the urine of infected animals. The clinical spectrum of the disease ranges from an influenza-like syndrome to Weil's disease and multiple organ dysfunction syndrome (1). The causative agents of human leptospirosis belong to the genus *Leptospira,* which contains both saprophytic and pathogenic species (1). *L. fainei* was first isolated from pigs in Australia (2). Subsequently, several reports based on serologic testing suggested that *L. fainei* might be pathogenic for humans as well (3,4). This potential has been recently confirmed by isolating *L. fainei* from the urine of two patients and the blood of one patient in Denmark (5). We report a typical case of leptospirosis in which *L. fainei* was directly observed in the cerebral spinal fluid (CSF) and was isolated from both CSF and blood.

## Case Report

In February 2001, a 39-year-old man was hospitalized in Marseilles, France, with fever and disorientation. This truck driver lived in Portugal and had been driving in Spain for 2 weeks before entering France. He arrived in France 3 days before admission. The day of admission, his co-workers found him confused and febrile after sleeping in his truck. He had no previous relevant medical history. On admission, his temperature was 40°C with tachycardia, and his blood pressure was 120/80 mm Hg. Clinical examination showed a drowsy, confused man with a bilateral headache, provoked myalgia of the legs, hepatalgia, and conjunctivitis. Neurologic examination showed signs of meningeal irritation, including cervical rigidity, Brudzinski's sign, hyperesthesia, and photophobia. There was no rash, and the rest of the physical examination was normal. Results of biochemical investigation included increased alanine aminotransferase (61 IU/L), aspartate aminotransferase (78 IU/L), lactate dehydrogenase (781 IU/L), fibrinogen level (6.44 g/L), and C-reactive protein (273 mg/L), associated with hypoglycemia (2.9 mmol/L), hypoalbuminemia (26 g/L), hypoproteinemia (54 g/L), and hypocholesterolemia (2.3 mmol/L). The leukocyte count was 11,000/mm^3^ with 69% polymorphonuclear forms. Thrombocytopenia (110 G/L) was also observed. The kaolin cephalin time was 58 s (control 34 s), and the prothrombin rate was 42%. Blood smears showed no parasites. Cerebral scanning was normal. Analysis of the CSF on day 1 showed 4 leukocytes/mm^3^, 50,000 erythrocytes/mm^3^, and elevated protein levels (1.48 g/L). On day 2, another CSF analysis showed 75 leukocytes/mm^3^, with 70% polymorphonuclear forms, 5,500 erythrocytes/mm^3^, and increased protein (0.64 g/L). Direct examination by dark-field microscopy of the CSF from day 2 was performed and controlled by two experimental investigators, who observed many spirochetes (6). The patient received a 10-day treatment with 12 g/day intravenous amoxicillin. The fever decreased to 38°C on day 4 and resolved on day 7. The patient was discharged from the hospital and remained well. No occupational or recreational risks for leptospirosis could be established. The patient had been traveling recently in Spain and Portugal but had no apparent exposure to sources of leptospires.

## The Study

**Figure 1:**
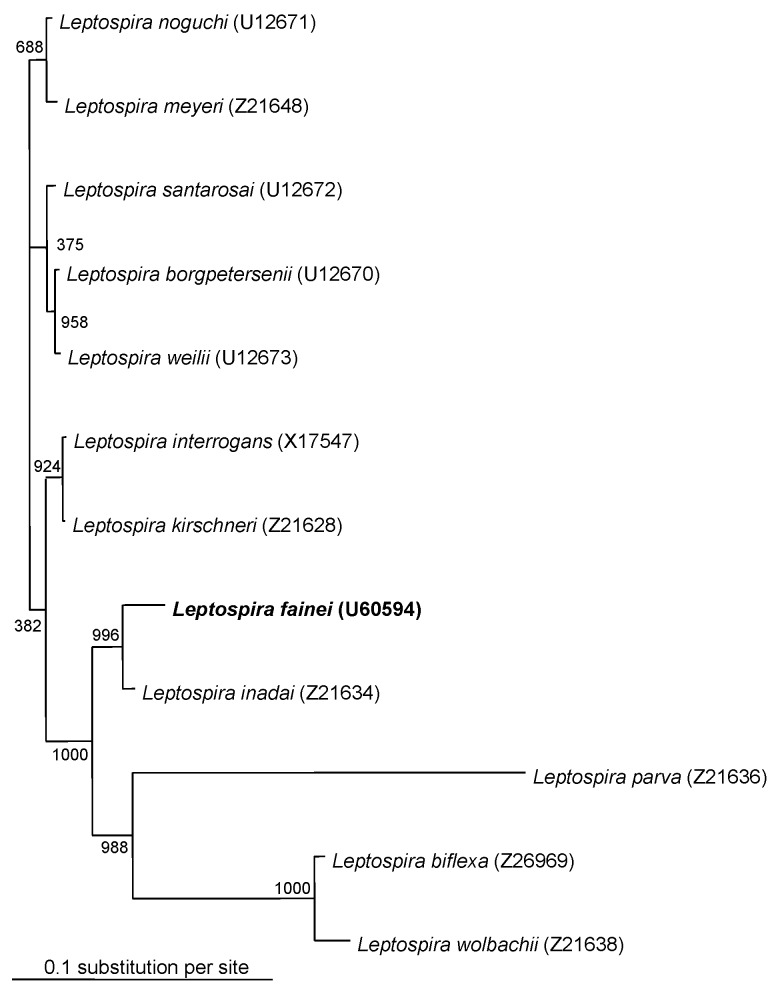
Dendogram representing phylogenetic relationships among members of the genus *Leptospira*. The tree was derived from a 1,295-bp fragment of the 16S rRNA gene and was constructed by the neighbor-joining method. Bootstrap values, expressed as a percentage of 1,000 replications, are given at the branching point. GenBank accession numbers are given in parentheses.

**Figure 2:**
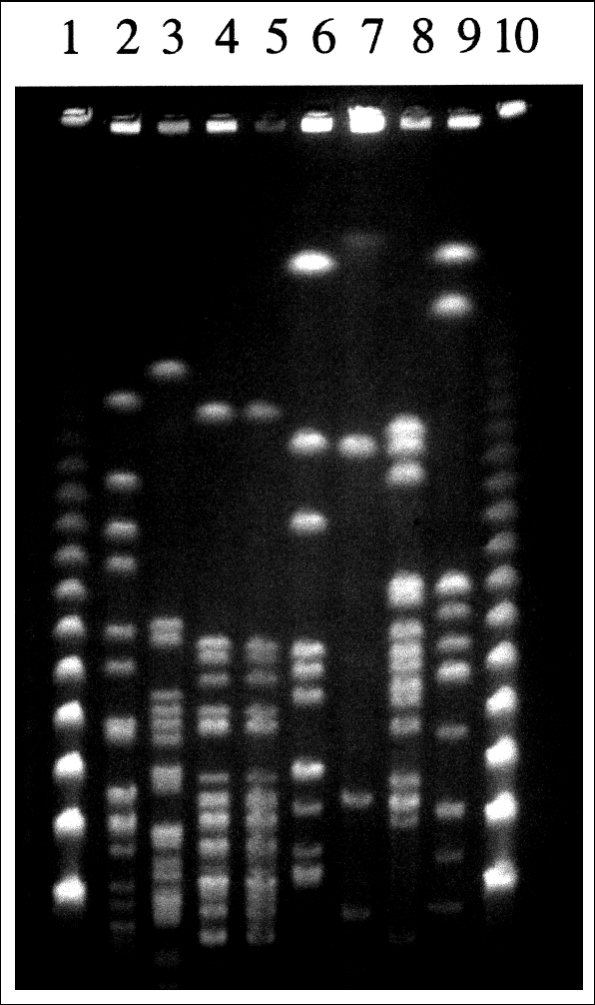
*Not*I restriction patterns of *Leptospira* strains obtained with the Bio-Rad apparatus (Richmond, CA) with a pulse time ramped from 5 to 90 s for 36 h. The lanes contain lambda concatemers (lanes 1 and 10) and DNA from isolates: patient strain (lane 2*)*; *L. fainei* hurstbridge, strain But 6 (lane 3); *L. inadai* Lyme, strain 10 (lane 4); *L. inadai* biflexa, strain LT430 (lane 5); *L. biflexa* patoc, strain Patoc I (lane 6); *L. meyeri* semaranga, strain VS173 (lane 7); *L. kirschneri* grippotyphosa, strain MoskvaV (lane 8); and *L. interrogans* icterohaemorrhagiae, strain Verdun (lane 9).

**Figure 3:**
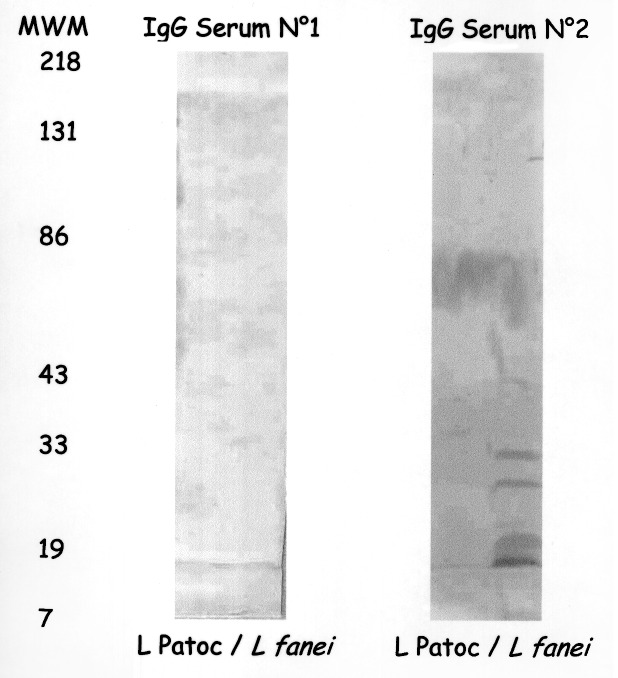
Western immunoblot of the patient's acute- (No. 1) and convalescent-phase (No. 2) sera on *Leptospira* serovar patoc and *L. fainei.* MWM indicates molecular weight markers.

Specific diagnostic tools were used to identify the spirochete responsible for this infection. Single 0.1 mL- and 0.01 mL-aliquots of lithium-heparin anticoagulated whole blood were spread onto 10 mL of *Leptospira* medium, a polysorbate medium similar to Ellinghausen and McCullough modified Johnson and Harris medium (EMJH) (Bio-Rad Laboratories, Inc., Aulnay/Bois, France), and was incubated at 30°C. The same procedure was carried out for CSF. For urine samples, the specimens were first filtered on 0.45- and then 0.22-µm filters before inoculation. Once a week, 10 µL of culture medium was examined by dark-field microscopy. Cultures from both blood and CSF were positive and yielded *Leptospira* after 1 week of incubation. Urine cultures remained negative. No agglutination of the isolated strain was obtained with any reference sera, except a weak agglutination (titer 400) with serovar hurstbridge antiserum. The genomic DNA of the spirochete was extracted from the blood culture by the Qiamp blood kit procedure (QIAGEN GmbH, Hilden, Germany). For polymerase chain reaction (PCR), universal 16S rDNA primers fD1 and rP2 (7) were used, and sequences of the PCR products were obtained as previously described (8). The 16S rDNA sequence of the isolate had 100% identity with the prototype strain sequence of *L. fainei* (GenBank accession no. 60594) (Figure 1). Pulsed-field gel electrophoresis of *Not*I macrorestriction fragments of *Leptospira* DNAs was performed as previously described (9). However, the *Not*I macrorestriction pattern was quite different from all patterns previously recorded for a large collection of *Leptospira* strains belonging to different species and serovars (Figure 2). Sera collected on days 4, 8, 10, and 45 were tested by a microimmunofluorescent antibody assay (MIFA) with *L. biflexa* serovar patoc, *L. fainei, L. interrogans,* and own strain as antigens (cut-off titer ≥200); Western immunoblot with *L. biflexa* patoc and *L. fainei* as antigens; and a microagglutination test (MAT) (10,11). For MAT, sera were incubated with suspensions of live leptospires belonging to 17 distinct serogroups, including a strain of *L. fainei* serovar hurstbridge. The titer was defined as the highest dilution giving 50% agglutination. The presence of immunoglobulin (Ig) M was investigated by an enzyme-linked immunosorbent assay (ELISA) developed at the Institut Pasteur, with serovar patoc as an antigen (12). All sera were negative by MIFA, MAT, and IgM ELISA, even when *L. fainei* was used as an antigen. For Western blot, the spirochetes were grown on *Leptospira* medium centrifuged and used at a concentration of 2 mg/mL. After blocking, the nitrocellulose was incubated overnight at 4°C with patient sera diluted 1:50. Seroconversion was demonstrated by Western blot analysis. The first two sera were negative by Western blot, but a reaction with *L. fainei* was observed in IgG and IgM at days 10 and 45. Sera reacted with protein bands of 28, 25, 24, and 19 kDa from *L. fainei*, while no reaction was obtained with serovar patoc (Figure 3).

## Conclusions

This clinical picture was highly suggestive of leptospirosis, with the association of meningeal syndrome, provoked leg myalgias, and conjunctivitis; nonspecific laboratory findings included hepatic enzyme elevation, hyperleukocytosis, thrombocytopenia, and low prothrombin rate (1). In fact, the case was considered clinically to be leptospirosis. The isolated *Leptospira* was identified as closely related to *L. fainei* on the basis of 16rRNA amplification and sequencing. The strain has been isolated in our laboratory in Marseille, where this species had never been cultivated. Thus, laboratory contamination is unlikely, and the isolated strain of *L. fainei,* which is close to the serovar hurstbridge*,* can be considered the causative agent of the patient’s meningitis. Moreover, a spirochete resembling a leptospire was seen by well-trained investigators at the dark-field microscope.

*L. fainei* has been only recently been considered an emerging pathogen. In Australia, a sample of 723 human sera from patients from a dairy and pig-producing area of Victoria, all of whom had symptoms consistent with leptospirosis, was submitted for leptospirosis serologic testing. The sera were also tested for antibodies to *L. fainei* serovar hurstbridge. MAT titers >128 were detected in 13.4% (3). Furthermore, a leptospirosis surveillance program was conducted for 12 months on the entire population of the Seychelles; the incidence of leptospirosis was 10/100,000, with a 20 % seroprevalence of *L. fainei*. This organism was suspected to be involved in severe forms such as acute renal failure, pulmonary hemorrhage, and possibly death (4).

The first human isolation of *L. fainei* occurred in Denmark, from two patients (5). The first patient had chronic disease with increasing jaundice for 6 months before admission to the hospital, and test results showed elevated hepatic enzymes. The second patient had abdominal and lower back pain for 5 months and severe headaches and dizziness for 2 months before admission. Thus, both patients had atypical chronic disease, unlike the typical case of leptospirosis that we describe.

In our case, we tested sera 4, 8, 10, and 45 days after onset of illness. No reactivity was detected by MIF and MAT tests, although a reaction in IgG and IgM at day 10 and 45 was detected by Western blot. Early antibiotic treatment may have affected the serologic responses. The two Danish patients from whom *L. fainei* was isolated were tested by MAT and showed no substantial serologic reaction (5). However, published serologic studies have shown that an immunologic response may be observed, in some cases at a high level (4). As reported here, Western blot may prove to be a good tool for diagnosis. Antibiotic treatment by amoxicillin (12 g/day for 10 days) was effective. In the Seychelles, for 8 patients among 75 with confirmed leptospirosis, a 5-day treatment with penicillin was insufficient to eradicate the bacteria, as indicated by positive PCR results (4). Treatment for longer than 1 week may be necessary, because *L. fainei* could be still isolated from Danish patients after a 1-week treatment with intravenous penicillin, but not after 4 weeks of amoxicillin treatment (3).

This case confirms the pathogenic role of *L. fainei* in humans and extends its geographic distribution to southern Europe. The clinical finding of our case did not differ from that of other *Leptospira* infections, but the route of exposure remains unknown. Although the usual procedures of direct detection, isolation, and identification of leptospires are effective for *L. fainei,* we did not observe substantial serologic reactivity. Further studies, including Western blot, are needed to explain the weak immune response and to evaluate the prevalence of infection by this newly discovered *Leptospira* species.
